# Mitochondrial dynamics regulates Drosophila intestinal stem cell differentiation

**DOI:** 10.1038/s41420-018-0083-0

**Published:** 2018-07-23

**Authors:** Hansong Deng, Shigeo Takashima, Manash Paul, Ming Guo, Volker Hartenstein

**Affiliations:** 1 Shanghai East Hospital, School of Life Sciences and Technology, Tongji University, Shanghai, 20092 China; 2 Department of Neurology, Department of Molecular and Medical Pharmacology, California Nanosystems Institute at UCLA David Geffen School of Medicine, University of California, Los Angeles, CA 90095 USA; 3 Department of Molecular Cell and Developmental Biology, University of California Los Angeles, Los Angeles, CA 90095 USA; 4 Present Address: Life Science Research Center, Gifu University, Gifu, 501-1194 Japan; 5 Present Address: Division of Pulmonary and Critical Care Medicine, David Geffen School of Medicine, University of California, Los Angeles, CA 90095 USA

**Keywords:** Cell proliferation, Organelles

## Abstract

Differentiation of stem/progenitor cells is associated with a substantial increase in mitochondrial mass and complexity. Mitochondrial dynamics, including the processes of fusion and fission, plays an important role for somatic cell reprogramming and pluripotency maintenance in induced pluripotent cells (iPSCs). However, the role of mitochondrial dynamics during stem/progenitor cell differentiation in vivo remains elusive. Here we found differentiation of Drosophila intestinal stem cell is accompanied with continuous mitochondrial fusion. Mitochondrial fusion defective(*opa1*RNAi) ISCs contain less mitochondrial membrane potential, reduced ATP, and increased ROS level. Surprisingly, suppressing fusion also resulted in the failure of progenitor cells to differentiate. Cells did not switch on the expression of differentiation markers, and instead continued to show characteristics of progenitor cells. Meanwhile, proliferation or apoptosis was unaffected. The differentiation defect could be rescued by concomitant inhibition of Drp1, a mitochondrial fission molecule. Moreover, ROS scavenger also partially rescues opa1RNAi-associated differentiation defects via down-regulating JNK activity. We propose that mitochondrial fusion plays a pivotal role in controlling the developmental switch of stem cell fate.

## Introduction

Stem differentiation is accompanied by pronounced changes in mitochondria. In embryonic stem cells (ESCs) or induced pluripotent stem cells (iPSCs), mitochondria are few in number, small and globular in shape, have fewer cristae, and are distributed as perinuclear clusters. During differentiation, mitochondria increase dramatically in mass and form an extensive tubular network^1–5^, while somatic cell reprogramming is accompanied by depletion of mitochondria through mitophagy (mitochondrial autophagy)^6^. For instance, cardio-myocyte differentiation in the embryonic heart is tightly linked to mitochondrial maturation^7^. Mouse hearts at embryonic day (E) 9.5 contain relatively few and immature mitochondria, characterized by rare and disorganized cristae. In E13.5 hearts the mitochondrial mass increases substantially, accompanied by maturation of the organelle as indicated by abundant laminar cristae^7,8^. Accordingly, one of the major obstacles for somatic reprogramming induced cardiomyocytes is to obtain functional mitochondria^8^. How these mitochondrial changes are regulated remains unexplored in vivo.

Mitochondrial morphology is regulated by continuous fusion and fission events. These dynamics processes are controlled by a group of GTPase proteins conserved from yeast to human. Mitofusins and opa1 are involved in outer and inner membrane fusion, respectively. Dynamin-related protein 1 (Drp1) is responsible for mitochondrial fission. Previous results from our lab and others showed that Drosophila homologs of fusion (MARF/mfn, opa1) and fission (drp1) are functionally conserved and are involved in pathogenesis of neuro-degeneration diseases^9–12^. Recent findings indicated that mitochondrial fission is required for stem cell pluripotency maintenance in iPSCs and ESCs^13^. Aside from affecting energy metabolism, mitochondrial fusion and fission has many other effects on developing and mature cells, in particular in regard to mitochondrial DNA (mtDNA) distribution, ROS production, calcium overload, and apoptosis^14–16^. However, the role of mitochondrial dynamics on stem cell differentiation in developing context is largely unknown.

**Fig. 1:**
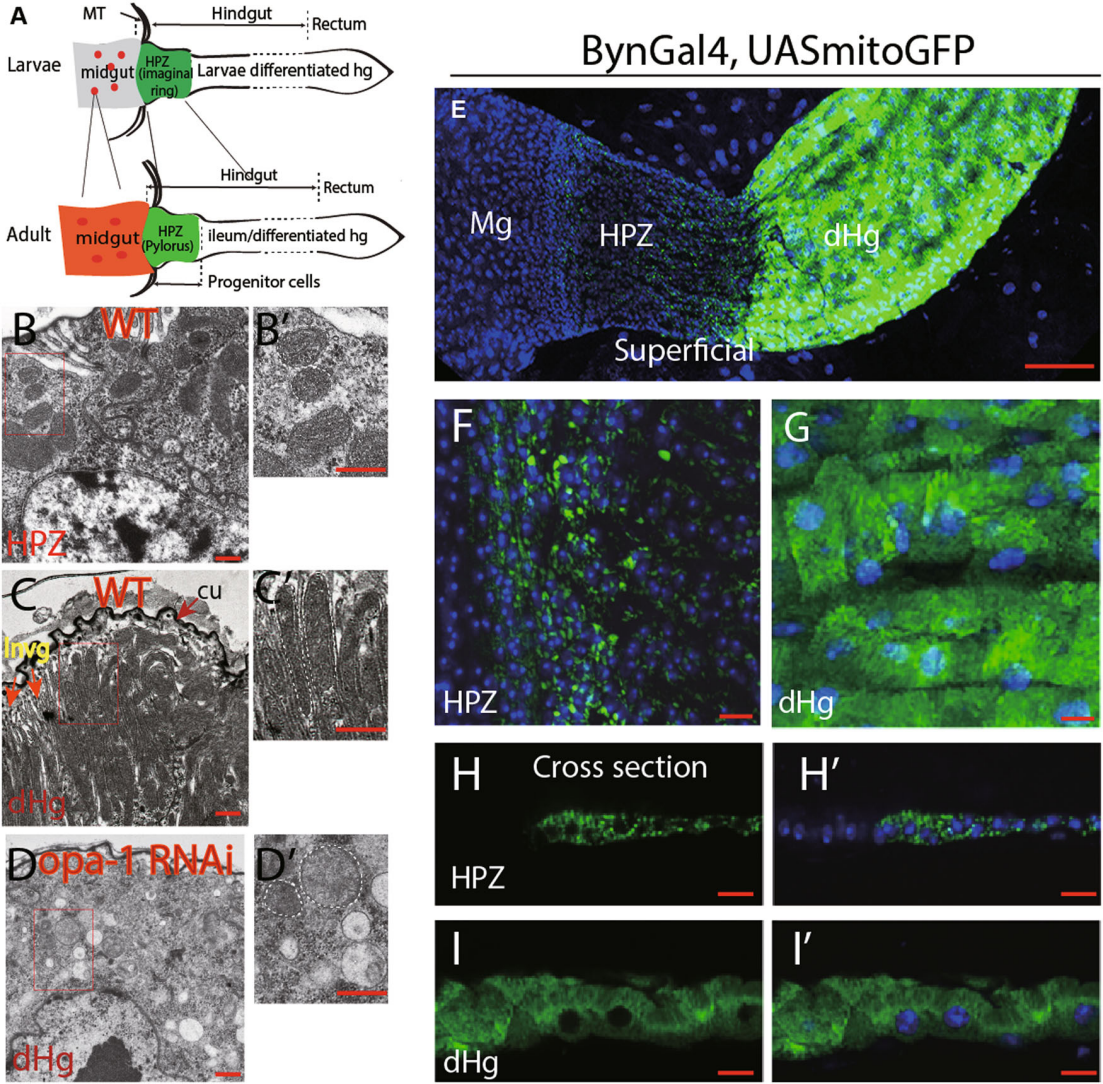
Extensive mitochondrial fusion during hindgut differentiation and *opa-1* RNAi inhibit the fusion process. **a** Schematic representation of gut development in Drosophila. Progenitors in the larvae stage give rise to the adult gut including its stem cell/progenitor populations. Midgut and hindgut are connected through Malpighian tubes (MT). During early metamorphosis, the progenitor populations expand over the larval gut, which undergoes programmed cell death. For instance, in the midgut, adult midgut progenitors (AMPs, denoted in red dots) in the larvae stage generate adult gut and stem cells (red dots). Similarly, hindgut proliferative zone (HPZ) cells (also called as hindgut imaginal ring, denoted in green) in the larvae stage generates the whole adult hindgut (green), including a subset of progenitors in the anterior hindgut (adult HPZ/Pylorus) and the differentiated hindgut (also called as ileum). Please refer to the main text for details. Mitochondria morphology of hindgut cells in different regions was observed by TEM from **b** to **d** and by confocal microscopy from **e** to **i**. **b** Mitochondria in adult HPZ cells. Note that the HPZ cells identity was based on the physical location and their unique morphology. **c** Mitochondria in adult differentiated cells. The matured enterocytes form a thick layer of cuticle structure (“cu” in brief) toward the lumen. Mitochondria aligned with membrane invigination (“invg” in brief). **d** Mitochondria in BynGal4>opa1 RNAi adult differentiated cells. For **b**–**d**, higher magnification of rectangle area shown on the right in **b**′–**d**′. Mitochondrial borders are marked with dashed lines. Cu cuticle, invg invigination, dHg differentiated hindgut, MT Malpighian tubes. **e**–**i** Mitochondria morphology visualized by *byn*-GAL4 > *UAS*-mito-GFP under confocal microscopy. **f** (HPZ domain) and **g** (differentiation hindgut, dHg) are higher magnification of **e**. **h**–**h**′ and **i**–**i**′ are cross-sectional view of HPZ and dHg cells, respectively. From **e** to **i**, TOTO3 labels nuclei in blue. Scale bars: 200 µm in **b**–**d**, 100 µm for **e**, 20 µm for **f**–**i**

The Drosophila adult gut and its stem cell populations derive from gut progenitors of the larval stage. In the midgut, these cells form small clusters (adult midgut progenitors or AMPs) scattered over the outer surface of the midgut epithelium^17,18^; in the hindgut, adult progenitors form a narrow domain, the hindgut proliferation zone (HPZ) also known as imaginal ring, located near the anterior hindgut boundary^19,20^ (Fig. 1a). During early metamorphosis, these progenitor populations expand over the larval gut which undergoes programmed cell death. Subsequently, most progenitors differentiate into adult enterocytes, whereas a small subpopulation of progenitors is held back and gives rise to adult progenitor/stem cells (Fig. 1a). These cells are quiescent in nature but are highly inducible upon tissue damage^19,21^. We speculated that mitochondrial dynamics and the functional changes they engender controls the switch from gut progenitor to differentiated enterocytes.

## Results

### Mitochondria undergo a dramatic increase in volume and complexity during differentiation of Drosophila intestinal cells

Mitochondria of hindgut cells were labeled by the *UAS*-mito-GFP transgene driven by *byn*-GAL4, a hindgut-specific driver^22,23^ (Fig. 1e–i and Fig. S1A). For the midgut, the *esg*-Gal4 driver was used^17^ (Fig. S2A and S2B). In the HPZ domain, the GFP-labeled mitochondria are few, small in volume, and perinuclear in location (Fig. 1e, f and h–h′). AMPs of the larva also possess small, granular mitochondria (Fig. S2A). Mitochondria of the differentiated hindgut and midgut enterocytes have strongly increased in volume and number (Fig. 1e, g, i–i′ and Fig. S2B). Transmission electron microscopy (TEM) was used to resolve mitochondrial morphology and structure at a higher-level resolution. The adult progenitor/stem cells in the HPZ domain located adjacent to the malpighian tubes and the mitochondria in these cells are small and round in shape and cristae is rarely observed (Fig. 1c). Mitochondria of midgut progenitors have similar dimensions (Fig. S2C). Differentiated adult hindgut enterocytes have enlarged in size and form a thick cuticle layer on the apical membrane facing the gut lumen; apical membranes are deeply invaginated, and mitochondria are located in between membrane invaginations (Fig. 1c and c′). Mitochondria are densely packed, elongated apico-basally, and often highly branched (Fig. 1c and c′). The size of mitochondria in these areas varies from 200 to 500 nm in diameter, and often several microns in length. Differentiated midgut enterocytes do not form cuticle, but a dense array of microvilli was formed at their apical pole. Mitochondria take up most of the cytoplasm of these cells; as in the hindgut, individual mitochondria are much larger, elongated, and often branched (Fig.S2D).

### Inhibiting mitochondrial fusion through opa1 or mfn knockdown causes hindgut stem/progenitor cells failed to differentiate

**Fig. 2:**
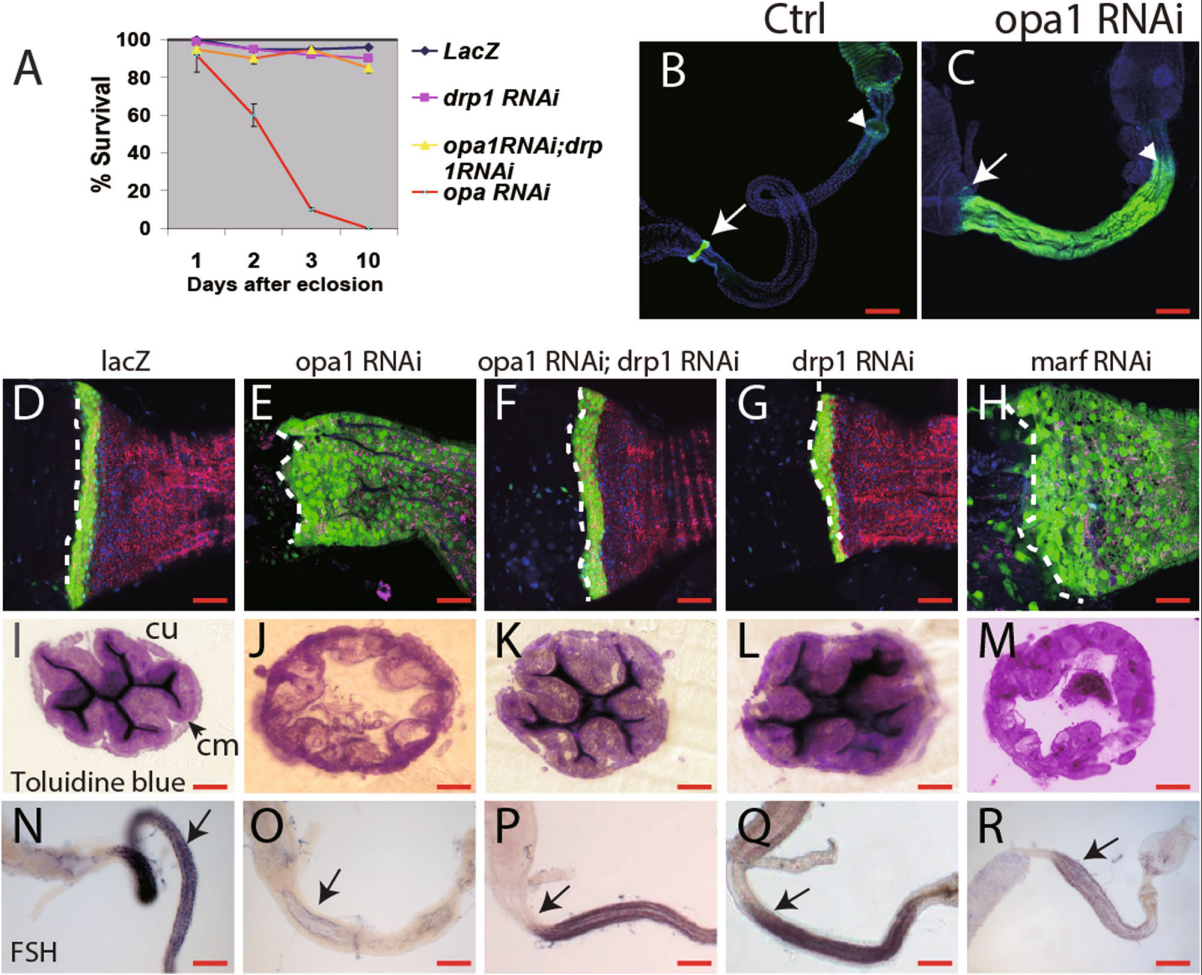
Enterocytes mis-differentiation induced by inhibiting mitochondrial fusion through *opa1* or *marf* RNAi in the hindgut can be rescued by *drp1* RNAi. **a** Survival curve of adult flies through knock down opa-1 (red), drp1 (green), both opa-1 and drp1 (yellow) or ctrl (blue) specifically in the hindgut by *byn*-GAL4. *Y-*axis is the survival percentage. *X-*axis is days after hatching out, at least 100 mated females counted for each genotype. **b,** **c** Short hindgut caused by *opa1* RNAi. Hindguts are highlighted between the arrow and arrowhead. Arrow marks the boundary of the midgut and the hindgut and the arrowhead marks the boundary of the hindgut and the rectum. Green signal is Stat-GFP. **d**–**h** The *Opa1*RNAi hindgut shows extensive expansion of a progenitor marker, Stat-GFP (green), cell membrane labeled by *myr-*RFP (red). The boundary between the midgut and the hindgut are outlined by dashed lines. Nuclei stained by TOTO3 in blue in all images. **i**–**m** Cellular structure of hindgut enterocytes stained by Toluidine blue. Circular muscle (“cm” in short) and cuticle (“cu” in short) are pointed in arrows. **n**–**r** In situ hybridization of FSH, a gene specifically transcribed in normal differentiated cells, as shown in **n**, dramatically decreased in *opa1* RNAi (**o**) or *marf* RNAi (**r**). FSH signal is largely restored by *drp1* RNAi (**p**). Scale bar for **b, c** and **n**–**r** is 200 µm, 40 µm for **d**–**m**

To manipulate mitochondrial dynamics, we took advantage of RNA interference (RNAi) and of over-expression of the responsible genes, using the UAS-GAL4 system and the temperature-sensitive GAL80 repressor (GAL80^ts^). *Byn*-Gal4-directed expression of an opa1-RNAi construct was able to block mitochondrial fusion in the hindgut. Mitochondria in opa1-RNAi-expressing hindgut enterocytes are ellipsoid in shape (Fig. 1d–d′ and Fig. S1C), a similar phenomenon was observed in *marf* RNAi hindguts (Fig. S1B). Aside from the expected defect in mitochondrial fusion, opa1-RNAi severely affected hindgut development. *Byn*-Gal4 > *opa-1* RNAi animals die within 2 days after eclosion (Fig. 2a), although the eclosion rate is comparable with the sibling controls (data not shown). Similar result was obtained when *opa1*RNAi was knocked down from the L1 stage using the GAL80^ts^ system, ruling out the possibility of embryogenesis mis-patterning. The hindgut is significantly shorter and slightly “fatter” compared with control flies (Fig. 2b, c).

Stat92E-GFP (stat-GFP in short), a reporter for JAK–STAT pathway activity ^24^whose expression is restricted to the HPZ in wild-type hindguts (Fig. 2d), remains strongly expressed throughout the entire hindgut of *opa-1* RNAi animals (Fig. 2e). Enterocytes is surrounded by basal circular muscles in the hindgut. The apical membrane inviginations and cuticles of enterocytes can be densely stained by Toluidine blue (Fig. 2i). However, the prospective enterocytes in *opa1* RNAi are highly dilated and no apical membrane invaginations or cuticle was formed inside (Fig. 1d, d′ and 2j). The acute lethality of opa1-RNA flies after eclosion and cellular structural abnormality in enterocytes suggested the lack of functionally differentiated cells. Indeed, *opa-1*(RNAi) hindguts lack signs of differentiation. By in situ hybridization, we found that FSH (CG7665), which is highly expressed in differentiated hindgut enterocytes of wild-type flies, is reduced or absent in *opa-1*(RNAi) flies (Fig. 2n, o). Similar phenomena were found in *byn*-GAL4>*marf* RNAi flies (Fig. 2h, m and r). To test whether an increase in mitochondrial fusion also causes hindgut dysfunction, we knocked down Drp1, an essential component of the mitochondrial fission machinery^25,26^. Expression of drp1-RNAi in the hindgut elicited a definitive change in the mito-GFP signal, suggesting irregular and enlarged mitochondria (Fig. S1D). However, the viability of drp1-RNAi animals is comparable to wild type (Fig. 2a). Cellular structure such as apical membrane invagination and cuticle as well as Stat-GFP expression are not significantly altered (Fig. 2g, l). Over-expression of the fusion gene Marf also produced no obvious defect on hindgut marker expression or cellular structure, although the mitochondria are more elongated than in control flies (data not shown). These results suggested that loss of fission or over-activation of fusion is dispensable for hindgut function. Next, we wanted to test if defects caused by *opa-1* RNAi and *marf* RNAi can be rescued by reduced fission (*drp1* RNAi) or over-fusion (*marf* OE). Indeed, the acute lethality of opa1-RNAi flies can be fully rescued by drp1 knockdown (Fig. 2a and data not shown). Importantly, the hindguts of the double knockdowns were properly elongated and expressed nearly normal pattern of Stat-GFP (Fig. 2f, k). Furthermore, enterocyte differentiation failure in the *opa1*RNAi hindgut can be restored by *drp1*RNAi: the hindgut of the double knockdowns expressed nearly normal level of FSH (Fig. 2p, q). Similar results were obtained by simultaneously knocking down Marf and drp1 (data not shown).

### Stem/progenitor cell proliferation is largely unaffected by *opa1*RNAi

**Fig. 3:**
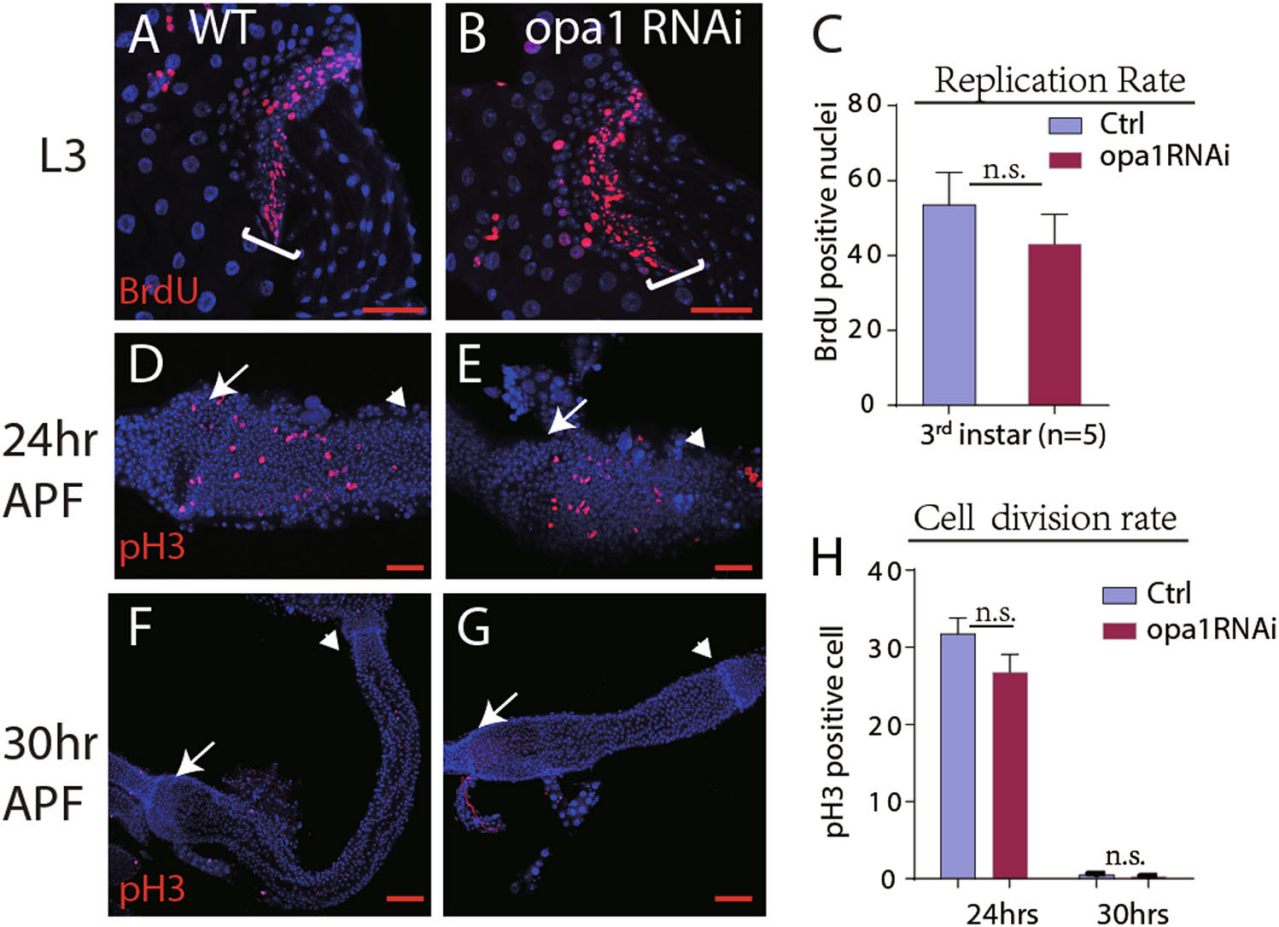
Stem/progenitor cell proliferation is largely unaffected by *opa1*RNAi. **a**–**c** Replication rate was measured by BrdU feeding in stage-matched third larvae. Representative images are shown as **a** (ctrl) and **b** (*opa1* RNAi). The HPZ zone is outlined with a white bracket. Quantification shown in **c**. No statistical significance was found, *n* = 5. **d**–**h** Proliferation rate was indicated by pH3-positive nuclei in larvae hindgut in 24 h APF (**d**, **e**) and 30 h APF (**f**, **g**). **d** and **f** are the Controls; **e** and **g** are *opa-1* RNAi larvae. Quantification shown in **h**. No statistical significance was found, *n* = 6. Error bars represent standard deviation (STDEV) in **c** and **h**. Arrows pointed to the boundary of the midgut and the hindgut, and TOTO3 stained nuclei in blue. Scale bar for 100 µm

As described above, the progenitor cell maker, Stat-GFP, expanded in the whole hindgut in *opa-1*(RNAi) and *marf* (RNAi) flies. We hypothesized that loss of mitochondrial fusion may trigger stem cell over-proliferation and form a stem cell like tumor. As a result, stem cells could fail to differentiate properly. To test this hypothesis, we analyzed proliferation by applying BrdU, which is specifically incorporated in DNA of S phase cells. Adult *opa-1*(RNAi) flies were fed BrdU-containing food for 24 h. After staining, we did not find a significant difference in the number of BrdU-containing cells between control and *opa-1*(RNAi) flies (data not shown). Proliferation was also tested during the larval stage. Mid-third instar larvae were raised on BrdU-containing food for 24 h. We found an average of 55 BrdU-positive cells in control larvae, and 50 in opa1-RNAi larvae; the difference is not significant (*n* = 6; Fig. 3a–c). Proliferation of the adult hindgut progenitors normally ceases around 24 h after puparium formation (apf)^27^. To rule out that the phase of proliferation is extended in *opa1*-RNAi, we monitored pupae 24 h apf and 30 h apf, using an antibody against phosphorylated histone 3 (pH3). At 24 h, *opa1-*RNAi hindguts showed the same number of mitotic cells as controls (Fig. 3d, e and h). Likewise, at 30 h apf, opa1-RNAi guts were devoid of mitotic cells, similar to controls (Fig. 3f–h). Wg acts as an essential signal to stimulate proliferation of the HPZ and at the same time inhibits differentiation^20^. Over-expression of Wg in the HPZ results in a hindgut phenotype that, in regard to lack of enterocyte differentiation, resembles the opa1-RNAi phenotype. To investigate whether knock down of opa1 affects wg expression, we used a Wg-lacZ reporter construct in the background of byn-Gal4-driven opa1-RNAi. No significant change in the pattern of Wg-lacZ expression is evident (Fig. S3A and S3B). Furthermore, CG31607, which normally transcribed only in HPZ cells, has no or slight expansion in *opa-1*(RNAi) flies (Fig. S3C and S3D). Taken together, these results suggest that the block of mitochondrial fusion by opa1-RNAi did not affect proliferation in the developing intestine.

### The hindgut defects in opa-1(RNAi) flies are not caused by apoptosis of differentiated enterocytes

Mitochondrial dynamics is closely related to apoptosis^14^, and we wondered whether the opa1 and marf knockdown phenotype is due to excessive cell death of enterocytes. Our findings speak against this hypothesis. No obvious increase of apoptotic cells was found in the *opa-1* RNAi hindgut by TUNEL staining (terminal deoxynucleotidyl transferase dUTP nick end labeling) (Fig. S4A and S4B). As a control, we found excessive TUNEL-positive cells when an rpr; hid over-expression construct was induced in the hindgut (Fig. S4C). Furthermore, over-expression of p35 and Diap, two caspase inhibitors^28^, failed to rescue the *opa-1*(RNAi) defects in terms of lethality or ectopic stat-GFP in the hindgut (Figures S4D–F and data not shown). These data suggest that the absence of differentiated cells in *opa-1*(RNAi) flies is due to a failure of differentiation, rather than apoptosis of differentiated cells.

### Mitochondrial defects induced by inhibiting mitochondrial fusion in hindgut

**Fig. 4:**
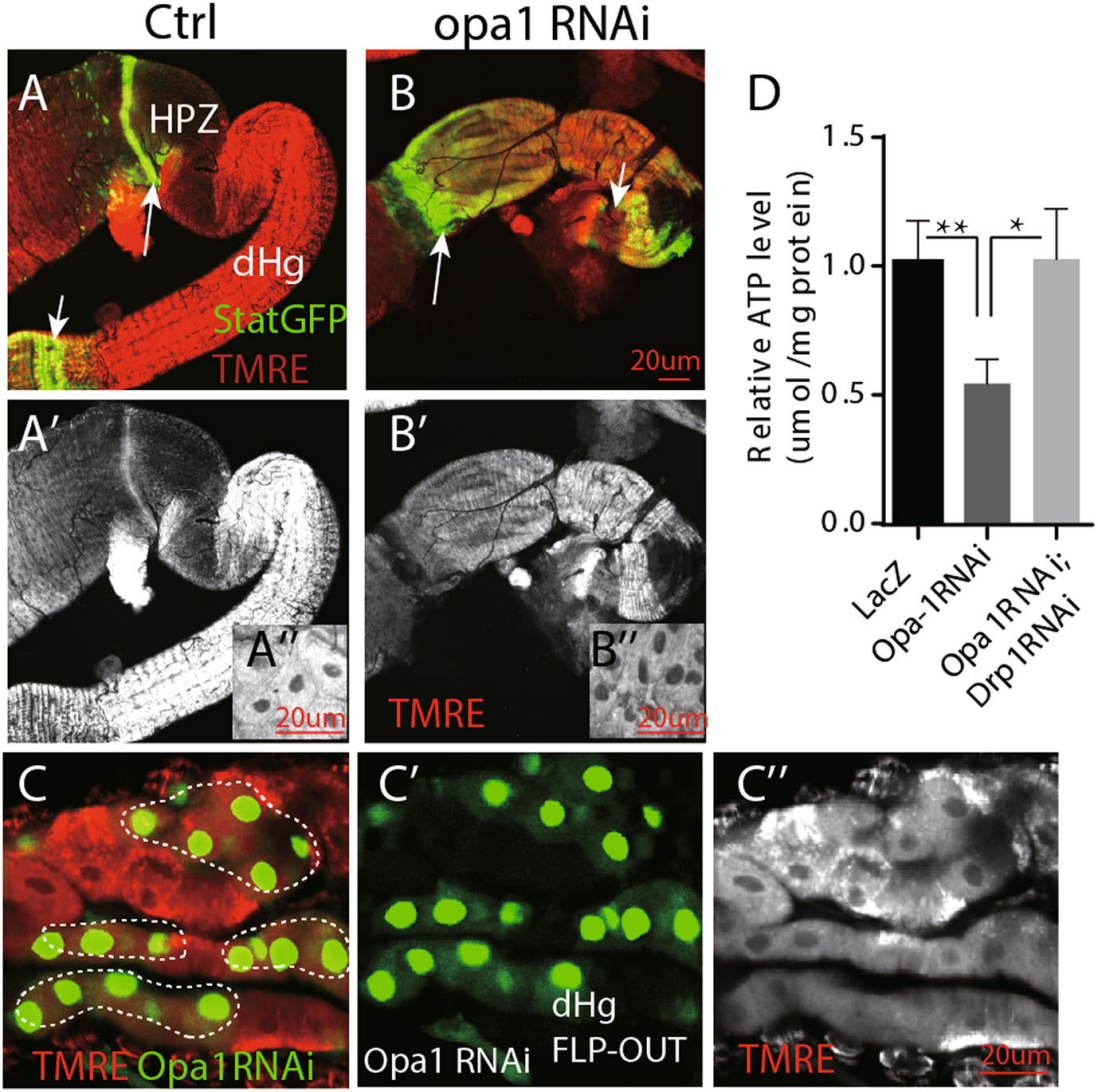
Mitochondrial defects induced by inhibiting mitochondrial fusion through *opa1*RNAi in hindgut. **a,** **b** TMRE staining in freshly dissected adult hindgut. Note wild-type enterocytes (“En” in short) have much higher fluorescent staining than cells in the HPZ domain. **a**′ and **b**′ are separate TMRE channels. **a‴** and **b‴** are higher magnified image of rectangle area in **a**′ and **b**′, respectively. The arrows point to the boundary of midgut and hindgut. The arrowheads denote the boundary of the hindgut and the rectum. **c** *Opa1* RNAi clones in the adult hindgut have much lower membrane potential by TMRE staining. Clones are marked by dashed lines. **c**′ and **c″** are separate GFP and TMRE channel, respectively. Genotype: hsFLP; *UAS*GFP; Tub < tub80 > GAL4/*opa1* RNAi. **d** Quantification of a relative ATP level of isolated hindguts with different genotypes: Ctrl, *opa1* RNAi, and *opa1* RNAi; *drp1* RNAi. Error bars represent standard deviation (STDEV). Scale bar is 20 µm

The functional output of mitochondria can be monitored by the mitochondrial membrane potential, which reflects the proton gradient generated by oxidative phosphorylation across the inner membrane. Tetramethylrhodamine ethyl ester (TMRE) is a commonly used dye to monitor mitochondria membrane potential in live cells^29^. We found that the increase in mitochondrial number and volume during enterocyte differentiation is accompanied by an increase in mitochondrial membrane potential. Hindgut enterocyte progenitors exhibit low TMRE signal, compared with the differentiated adult cells (Fig. 4a/a′/a″ and Fig. S5A–C). Knocking-down opa1 caused a reduced functional output of mitochondria. The membrane potential dropped to a level that, quantitatively, corresponded to that of enterocyte progenitors (compare panels Fig. 4a′/a″ and b′/b″). Significant decrease of membrane potential was also found in *opa1* RNAi clones (Fig. 4c/c′/c″). Mitochondrial ATP production is coupled with membrane potential^30^. As expected, *opa1* RNAi hindguts have significantly lower ATP level than controls. Although drp1 knocking down also decrease ATP production slightly, *drp1* RNAi significantly restores ATP level production of *opa1* RNAi hindguts (Fig. 4d).

### Excessive ROS induced JNK activity contributes to mis-differentiation in opa1RNAi hindgut

We and others previously showed that ROS function as a signal molecule controls stem cell proliferation in Drosophila ISCs and mammalian airway basal stem cells (ABSCs)^31,32^. Since most of ROS are produced by mitochondria and are closely regulated by mitochondrial fission^14^. We sought to test whether ROS is involved in hindgut defects of *opa1*RNAi flies.

**Fig. 5:**
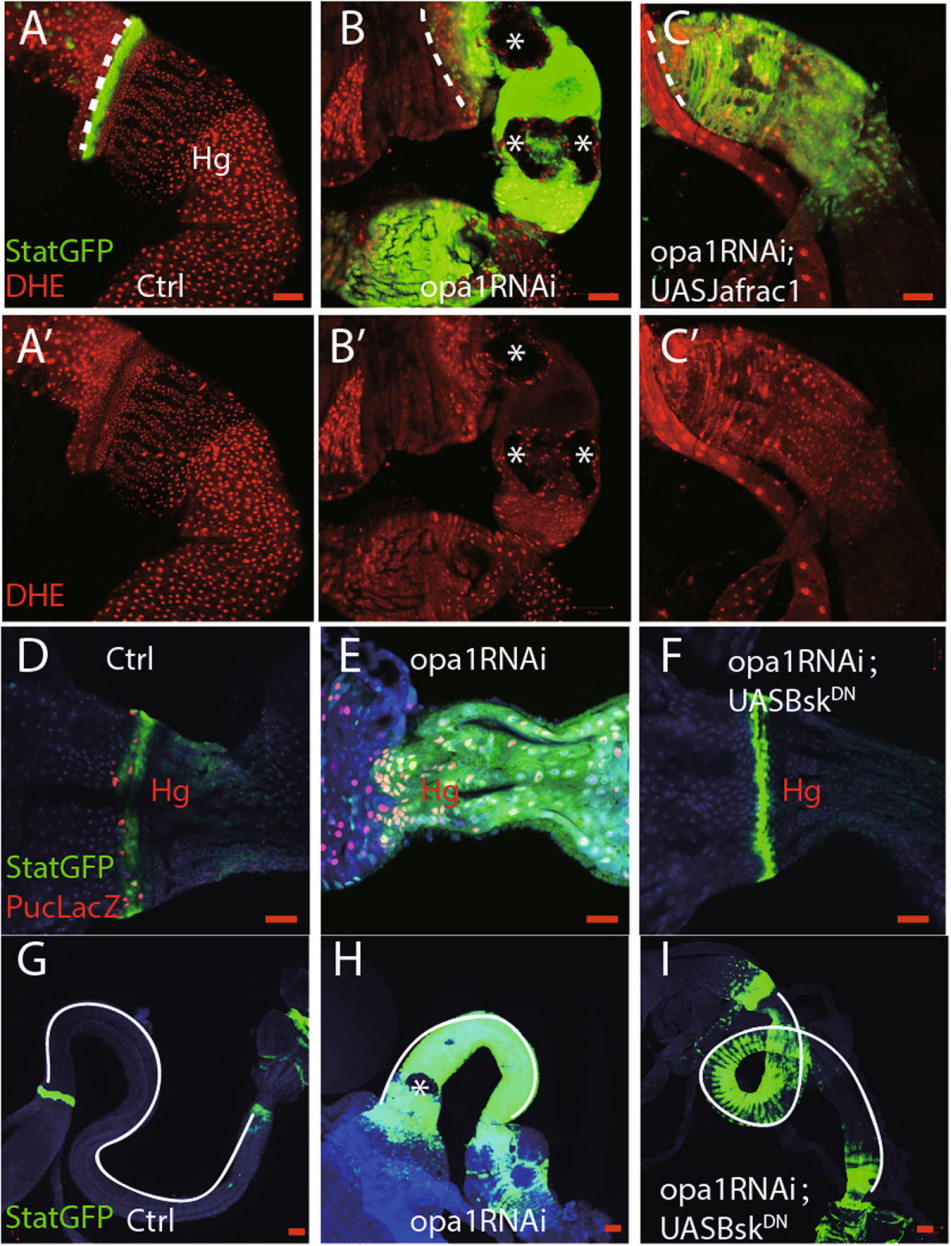
ROS–JNK pathway contribute to differentiation defects in *opa1*RNAi hindguts. **a**–**c** DHE staining of freshly dissected hindgut. **a**′–**c**′ are DHE channel. Asterisk denotes potentially non-apoptotic cell death in *opa1*RNAi hindguts. **d**–**f** Anti-beta Gal staining against Puc-LacZ in different genotypes. **g**–**i** Differentiation index, such as Stat::GFP and gut length, was compared in different conditions. Gut length was delineated out by white lines. Scale bar is 20 µm

Dihydroethidium (DHE) was used as a specific dye to measure the ROS level in freshly dissected guts. DHE fluorescence is relatively lower in the HPZ zone, while higher in differentiated cells in control hindguts (Figs. 5a and a′). However, in opa1-RNAi hindguts, all hindgut cells have a relatively higher ROS level (Note: ‘black holes’ likely due to non-apoptotic cell death in *opa1*RNAi hindguts, shown as asterisk in Fig. 5b and b′). It implies that elevated ROS production may cause differentiation defects in *opa1*RNAi hindguts. As expected, overexpressing Jafrac1, a thioredoxin peroxidase 1, led to a significantly reduction of ROS level and suppressed non-apoptotic cell death phenotype in *opa1*RNAi hindguts (Fig.5c and c′).

JNK mediated multiple downstream effects of ROS signaling^31,33^. Puc-LacZ was used as a reporter for JNK activity^33^. We found that *opa1*RNAi hindguts have much higher Puc-LacZ staining than controls and can be fully suppressed by overexpressing a dominant-negative form of JNK, UASBsk^DN^ (Fig. 5d, f). Moreover, UASBsk^DN^ can partially suppress *opa1*RNAi-associated differentiation defects, such as StatGFP expansion and gut length (Fig. 5g–i).

All these results indicated that the ROS–JNK pathway participated in *opa1*RNAi hindgut defects.

### Inhibit mitochondrial fission also inhibit midgut enterocyte differentiation

**Fig. 6:**
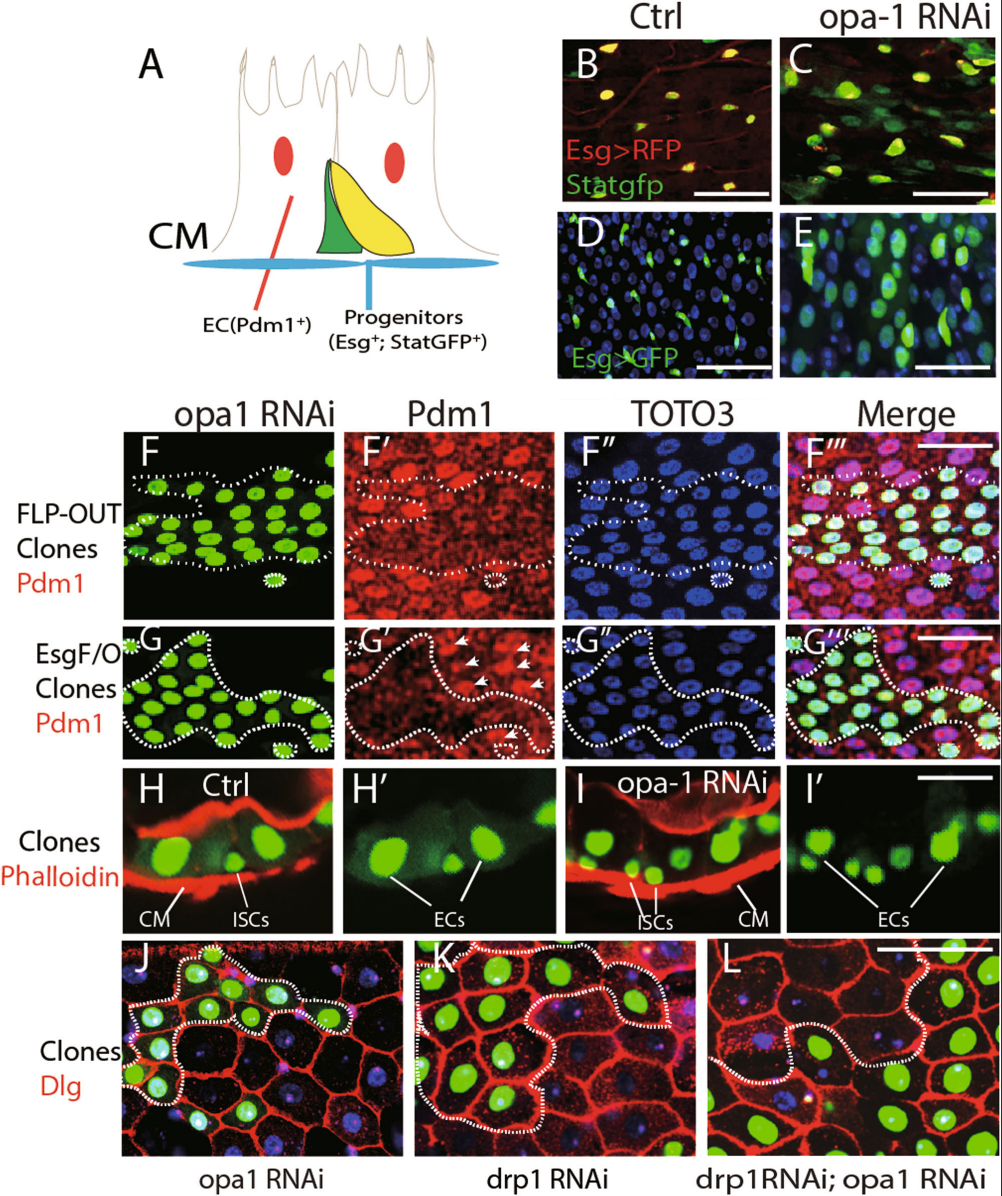
Inhibiting mitochondrial fission also impairs midgut enterocyte differentiation. **a** Schematic view of cell types in the adult posterior midgut. Basal located progenitor cells (green) are *Esg* and Stat-GFP positive. Enlarged enterocytes can be labeled by differentiation marker Pdm1. CM is the brief for circular muscle. Both circular muscle and actin enriched microvilli in enterocytes can be stained by Phalloidin. **b**, **c** Ectopic Stat-GFP-positive cells (green) in the *opa-1* RNAi midgut. The progenitor cells in the normal midgut are labeled with membrane-bound UAS-*myr-*RFP driven by *Esg*-GAL4. **d**, **e** Massive enterocyte-like cells are Esg positive (green) in the *opa-1* RNAi midgut. In normal midgut, Esg-positive diploid cells are labeled by *esg*-GAL4; *UAS*GFP (**d**). **f** *Opa-1* RNAi Flp-out clones induced in the larvae stage have less Pdm-1 expression in the adult midgut. GFP-positive clones are outlined with dashed lines. **f**, **f′**, **f″**, and **f‴** are GFP, Pdm1, TOTO3, and Merged channel, respectively. **g** *Opa-1* RNAi clones induced in the adult stage have less Pdm-1 expression in the adult midgut. GFP-positive clones are marked with dashed lines. **g**, **g′**, **g″**, and **g‴** are GFP, Pdm1, TOTO3, and Merged channel, respectively. **h,** **i** Mis-differentiation of enterocytes in *opa1* RNAi clones. Circular muscle is “CM” in short. Actin enriched microvilli and circular muscles are stained with phalloidin in red. **h′** and **i′** are GFP channel of **h** and **i,** respectively. **j**–**l** Enterocytes in *opa1* RNAi clones are smaller in size. GFP-positive clones are labeled with dashed lines. The cell boundary was stained with Dlg in red. TOTO3 labels nuclei in blue. Scale bar is 20 µm. Genotype for **f** and **i**: hsFLP; *UAS*GFP; Tub < tub80 > GAL4/*opa1* RNAi. Genotype for **g**: *hs*FLP; *esg*-GAL4, *UAS*GFP, TubGAL80;^ts^ Tub < tub80 > GAL4/*opa1* RNAi Genotype for **h**: *hs*FLP; *UAS*GFP; Tub < tub80 > GAL4 Genotype for **k**: *hs*FLP; *UAS*GFP; Tub < tub80 > GAL4/*drp1* RNAi Genotype for **l**: *hs*FLP; *UAS*GFP; Tub < tub80 > GAL4/*drp1* RNAi, *opa1* RNAi

Next, we examined the requirement of fusion for adult midgut differentiation. Larvae expressing *opa1* RNAi under the *esg*-GAL4 driver from the early (L1) stage onward fail to reach the pupal stage and arrest at the third instar or pre-pupal stage (data not shown), probably because of the multiple-tissue expression of *esg*-GAL4 which may cause pleiotropic defects in development^34^. Differentiation defects was observed in the midgut progenitors of these animals as well. In wild type, midgut progenitors split into two cell types. The center of each progenitor cluster is occupied by small, rounded midgut progenitors which will form the adult midgut. These cells are surrounded by large, flattened peripheral cells which during early metamorphosis differentiate into a transient pupal midgut^35,36^ (Fig. S6A and B). Following opa1-RNAi expression in midgut progenitors, peripheral cells did not differentiate (Fig. S6C). However, drp1 knocking down fully suppressed the differentiation defects (Fig. S6D and E). To overcome the early lethality, we expressed *opa1* RNAi from the late third instar stage. Under these conditions, larvae can pupate and eclose. Examination of midguts of freshly eclosed adults revealed a phenotype that resembled in many aspects of the above described hindgut phenotype. A large proportion of midgut cells shows signs of immaturity, in terms of small size, continued expression of esg, and Stat-GFP, another ISC/EB marker^35,37^(Fig. 6a–e). Moreover, the expression of Delta also showed massive expansion along with Esg in *opa1* RNAi flies (Fig. S7A, B). On the other hand, Pdm-1, a differentiation marker for enterocyte^38,39^, was normally expressed in most of these cells (data not shown). We speculated that the relatively late onset of *opa1* RNAi expression (see above) may in part be responsible for the weak differentiation phenotype, compared to the hindgut. We therefore examined the effect of *opa1* knock-down FLP-out clones. First instar larvae, with the genotype of *hs*FLP; tub > GAL80 > GAL4, *UAS*-GFP/*UAS*-*opa1*RNAi, were heat-shocked at 37 °C for 1 h to induce *opa1* RNAi-expressing clones and the phenotype was examined at the adult stage. Clones of cells knocked-down for opa1 had no or decreased level of the differentiation marker Pdm-1, compared with clones without *opa-1* RNAi (Fig. 6f–f‴). Meanwhile, clones bearing multiple copies of *opa1*RNAi induced in adult by another lineage tracing stock (esg^ts^GFP; *UAS*-FLP, tub < CD2 > GAL4) also showed smaller and significantly weaker expression of Pdm1 (Fig. 6g–g‴). Matured enterocytes often migrated toward the apical gut lumen and form actin enriched microvilli, which was stained by phalloidin. In control GFP-positive clones, enterocytes are adjacent to the phalloidin-stained structure (Fig. 6h); however, the enterocytes retained in the basal side in opa1 RNAi clones (Fig. 6i). Finally, the cell size in the *opa1*RNAi clones is much smaller compared with the adjacent non-labeled differentiated cells (Fig. 6j), indicating the immature differentiation. Again, this defect can be rescued by drp1 knock-down (Fig. 6k, l).

## Discussion

**Fig. 7:**
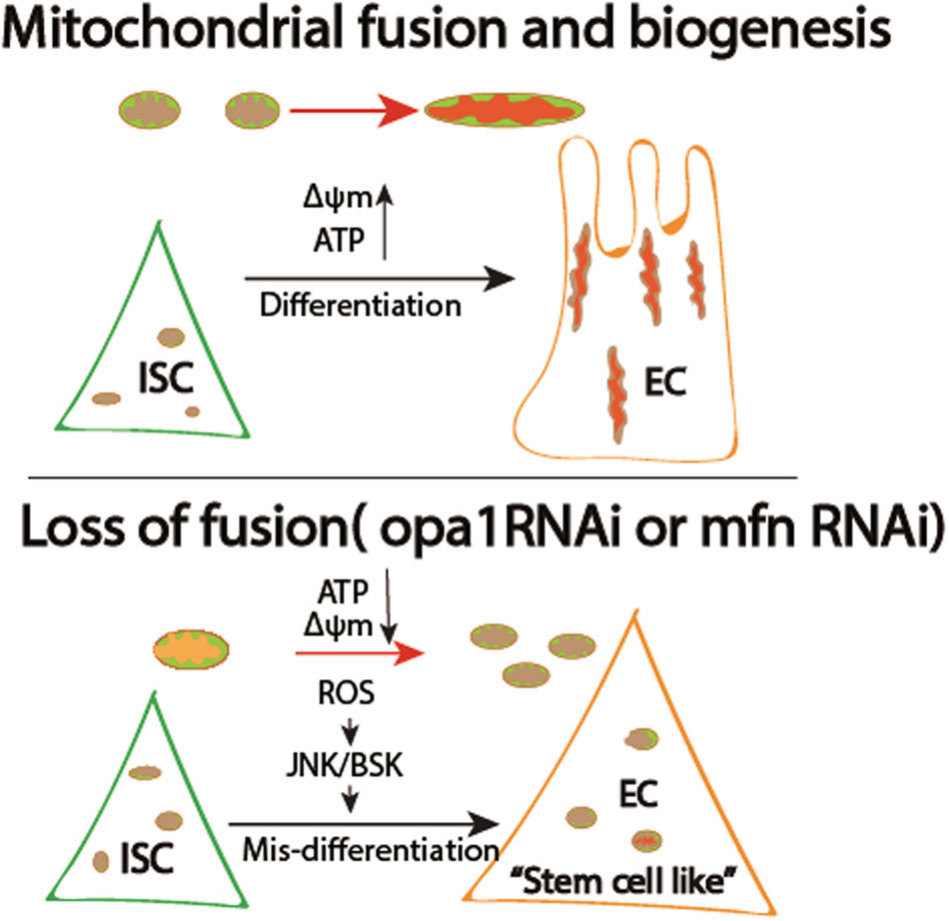
Model on mitochondrial fission-mediated differentiation failure in Drosophila intestine stem cells. Proliferative cells, including Drosophila intestine stem cells in this scenario, bearing hypo-active, smaller and fewer mitochondria with having a relative lower membrane potential (marked in purple). Hence, self-renewal is largely independent on mitochondria function. During differentiation, mitochondria continuously undergo fusion and biogenesis to cope with the energy demand. Correspondingly, mitochondria became more active (marked in bright red). Meanwhile, mitochondria may send some retrograde signals, such as ROS, ATP, and/or some unknown molecules back to nucleus to program the differentiation process. On the other hand, loss of fusion by opa-1 RNAi or marf RNAi causes mis-differentiation of progenitor cells. The “prospective” differentiated cells are much smaller and still express progenitor marker (statGFP) but not differentiated marker (FSH). Loss of fusion can block production

Our results here indicated that mitochondrial fusion, followed by increased functional output, forms part of the causal chain that switches the cells state from stem/progenitor cell towards differentiation. Thus, preventing fusion by knock-down of opa-1 or marf can block enterocyte differentiation in intestinal stem/progenitor cells. On the other hand, blocking fission by over-expression of marf or knock down drp1 did not cause obvious defects in differentiation. Moreover, blocking fission by knocking down of drp-1 rescued the defects of opa-1 RNAi. We then demonstrated that mitochondria in the opa1 RNAi hindgut have lower membrane potential, less ATP production, and higher ROS. We then showed that a high ROS level contribute to mis-differentiation in opa1RNAi hindgut by increasing JNK activity. These findings indicate that mitochondrial fusion is critical for enterocyte differentiation (Fig. 7).

ROS, in conjunction with the JNK pathway, affects stem cell activity, cell fate transition and cell survival^40^. Our previous results showed that in mouse and human ABSCs, low to moderate ROS levels is required for stem cell self-renewal and proliferation^31^. Similarly, Drosophila midgut stem cells have relatively lower ROS level compared with differentiated enterocytes and increasing ROS level promotes proliferation rate^32^. Here, we showed that ROS-mediated JNK pathway contributed to *opa1*RNAi hindgut mis-differentiation, indicating intrinsic difference of stem cells in response to ROS signaling. Besides ROS, mitochondrial fusion could also trigger retrograde signaling pathways such as calcium^41^, which we recently described to capable to alter stem cell activity in Drosophila midgut ISCs^42^

In addition to these retrograde signaling processes, mitochondrial fusion might also cause energetic changes (ATP level) that affect differentiation. Although genetically and pharmacologically augment of cellular ATP level fail to rescue the mis-differentiation in opa1 RNAi flies (data not shown), we cannot exclude the possibility that optimal cellular level of ATP regulates stem cell differentiation.

Taken together, we described that the net effect of mitochondrial dynamics in Drosophila intestine stem cell lineage is a progressive fusion process and this process is essential for stem cell differentiation. Considering mitochondrial maturation is also crucial for iPSCs differentiation, our results suggested that boosting mitochondrial fusion could produce more functionally differentiated cells and can be targeted for regenerative medicine.

## Materials and methods

### Fly stocks and genetics

Flies used in this study were (donors in parentheses) *byn*-Gal4 (J. Lengyel), *hh*-Gal4 (K. Basler), *esg*-GAL4 (N. Perrimon) *UAS*-P35, *UAS-*Diap (B. Hay), Esg^ts^GFP (B. Edgar), 10xStat92E-GFP (E. Bach), *UAS*Jafrac1(H. Jasper) and the following stocks were obtained from Bloomington stock center: tub-GAL80^ts^, Ser-GAL4, *UAS*-*mito*-GFP, *Hs*FLP, Aygal4, UASGFP or from the National Institute of Genetics, Japan: *Wg-lacZ*/CyO. All flies were reared with normal fly food at room temperature or in incubators at 18 °C, 25 °C, or 29 °C. FLP-OUT clones were induced at late second instar or early third instar larvae by heat shock 1 h at 37 °C water bath. Clones are marked with GFP. For hindgut differentiation experiments, early second instar larvae were shift from 18 °C –22 °C to 29 °C. The hindguts were dissected 2 days after hatching out.

### Transgene construct

UASsmDRP1: To silence DRP1, coding region in the DRP1 was targeted using a microRNA-based technology^43^. PCR products of an microRNA precursor was cloned into pUAST. All cloned PCR products were confirmed by DNA sequencing^43^.

### Survival assay

Around 100 mated female flies for each genotype were counted, which were raised and kept in 29 °C from first larvae on. Dead flies were scored each day in 2 weeks. Three independent experiments were performed.

### BrdU incorporation assay

Labelling proliferative cells with BrdU was performed by feeding adult flies or larvae with BrdU. Adult flies of 1 week after eclosion or older were reared with normal fly food containing BrdU (1 mg/ml, Sigma) for 3 or 7 days consecutively. Third instar larvae were fed with the same food containing BrdU for 2–4 h. Hindguts of adults or larvae were dissected and fixed immediately after BrdU labelling or subsequently reared with normal fly food without BrdU for another 7–14 days before dissection, and then treated with 2 N HCl for 30 min on ice, before being processed for antibody staining.

### Toluidine blue staining and TEM

The hindgut was excised under a dissection microscope, fixed with 2.5% glutaraldehyde in 0.05 M phosphate buffer (pH 7.4) for 1.5 h at 4 °C, and washed three times with 0.05 M phosphate buffer at 4 °C, post-fixed with 1.0% OsO_4_ for 1 h at 4 °C, washed with phosphate buffer, dehydrated in ethanol, and embedded in Spurr resin. Thick section was stained with Toluidine blue and ultra-thin sections of the embedded guts were double-stained with uranyl acetate and lead citrate and examined with a JEOL 100C transmission electron microscope (TEM).

### In situ hybridization

In situ hybridization of FSH is followed as described^44^. Briefly, dissected hindguts were fixed with 4% formaldehyde and stored in 100% methanol at −20 °C until use. The hindguts were rehydrated with a descending methanol series and treated with PBS containing 0.1% Tween 20 (PBS-tw). They were then reacted with 10 μg/mL of proteinase K diluted with PBS-tw; the reaction was stopped with 2 mg/mL of glycine dissolved in PBS-tw. After being fixed with 4% formaldehyde again and washed with PBS-tw, hindguts were hybridized with a digoxygenin-labeled RNA probe prepared against FSH or CG31607 cDNA diluted with hybridization buffer at 60 °C for overnight. After washing out non-hybridized probes with 50% formamide diluted with 10× SSCT, 2× SSCT, and 0.2× SSCT (performed at 60 °C) and then briefly washed with PBS-tw, they were blocked with 0.2% Blocking reagent (Roche, Indianapolis, IN) diluted with PBS-tw and then treated with anti-Digoxygenin antibody labeled with alkaline-phosphatase (Roche) overnight. After washing with PBS-tw, the hybridized probe was detected by NBT/BCIP (tablet, Roche). The primer sequence of FSH is: CACGGGGCTGAAGGTCTACGGAT and TGCAGCCAAGCCCGTACTGCCAA.

CG31607 is now called CG43394 according to the FlyBase and its probe was synthesized from a plasmid obtained from DGRC and the plasmid # is RE17733.

### Antibody staining

Hindguts and midgut were fixed with 4% formaldehyde/PBT for 45 min at room temperature and then treated with primary antibody overnight at 4 °C. After treatment with secondary antibody conjugated with fluorescent dye, hindguts and midguts were mounted with Vectashield (Sigma). A nuclear indicator (TOTO3) was added to the mounting medium if necessary (1:2000 dilutions). Antibodies used in this study were: mouse anti-Arm (1:10; DSHB, University of Iowa), mouse anti-β-gal (1:100; Promega), Mouse anti-CycA, mouse anti-CycB (1:5; DSHB), mouse anti-LaminC (1:50; DSHB), Rabbit Phosho-H3 (1:100; Molecular Probes), Mouse anti-Delta (1:50; DSHB), and rabbit anti-Pdm1 (gift from Xiaohang Yang, Institute of Molecular and Cell Biology, Singapore).

### Mitochondria membrane potential measurement

For membrane potential, whole gut was dissected in Schneider solution at room temperature, then stained in Schneider solution containing 1 μM TMRE (dissolved in 100% ethanol, from Molecular Probes) for 20 min, washed in Schneider 2 × 5 min, and then, directly imaged under a confocal microscope. For ROS staining, DHE staining was followed as described^32^. In brief, fresh guts were dissected and incubated in Schneider medium containing 50 μM DHE (dissolved in DMSO, from Molecular Probes) for 20 min, washed in Schneider 2 × 5 min, and then, directly imaged under a confocal microscope. All these experiments were carried out in dark.

### ATP measurement

The hindgut ATP level was measured using a luciferase-based bioluminescence assay (ATP Bioluminescence Assay Kit HS II; Roche Applied Science). For each measurement, ten hindguts were freshly dissected out (with midgut removed) and immediately homogenized in 50 µl lysis buffer. The lysate was boiled for 5 min and cleared by centrifugation at 20,000 *g* for 1 min. Five microliters of cleared lysate was added to 90 µl dilution buffer and 5 µl luciferase, and the luminescence was immediately measured using a 96-well plate luminometer. Each reading was converted to the amount of ATP per hindgut based on the standard curve generated with ATP standards. The readings will then be normalized with the protein level measured by BCA Bradford assay. Three measurements were made for each genotype.

## Electronic supplementary material

Supplemental information
