# Supplementary material for: Mitochondrial dynamics regulates Drosophila intestinal stem cell differentiation
Source: Cell Death Discov. 2018 Jul 23;4:81. doi: 10.1038/s41420-018-0083-0 (PMC6056485; doi:10.1038/s41420-018-0083-0)
Supplement: Supplementary file 1 — Supplemental information [file 41420_2018_83_MOESM1_ESM.docx]

**Supplemental information**

**Figure S1. Mitochondrial morphology changes during hindgut differentiation process**

Mitochondria morphology was visualized by *UAS*-mito-GFP driven by *byn*-GAL4. Nuclei stained by TOTO-3 In red in all images. Scale bar: 5µm.


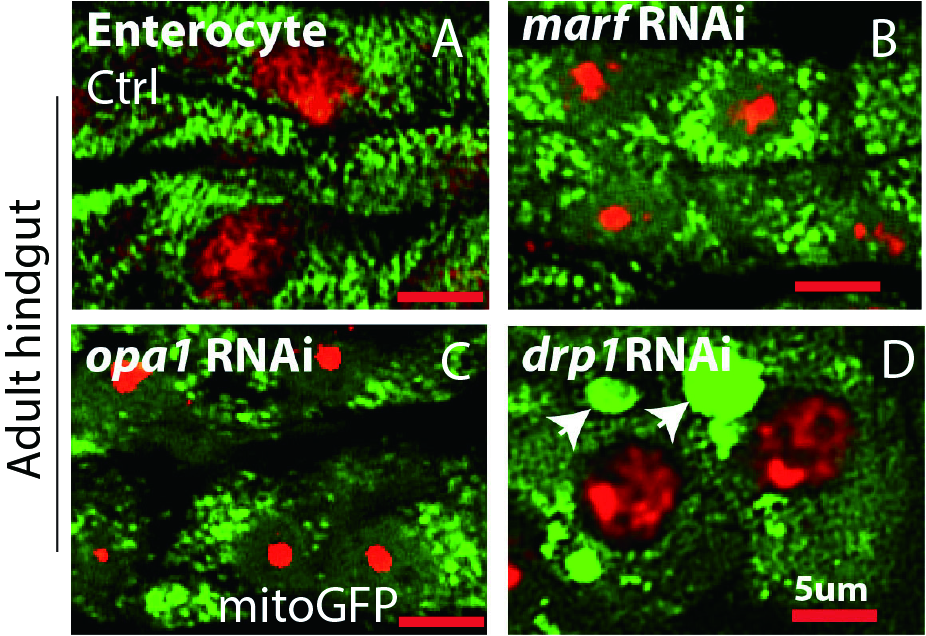


**Figure S2. Mitochondria morphology changes during midgut differentiation.**

(A) Mitochondria in larvae AMP cell cluster. Mitochondria morphology was visualized by *UAS*-mito-GFP driven by *esg*-GAL4. A’ is the mitochondria channel.

(B) Mitochondria in adult differentiated enterocytes. Mitochondria morphology was visualized by *UAS*-mito-GFP driven by *tub*-GAL4. B’ is the mitochondria channel only.

(C) Mitochondria morphology in AMP cells under TEM microscopy. Arrows point to mitochondria.

(D) Mitochondria morphology in adult enterocytes under TEM microscopy. Arrows point to mitochondria. Nuclei are highlighted in light blue color in C and D. Nuclei are counter stained by TOTO-3 in red in A and B. Scale bar: A-B, 20µm, C-D 5µm.


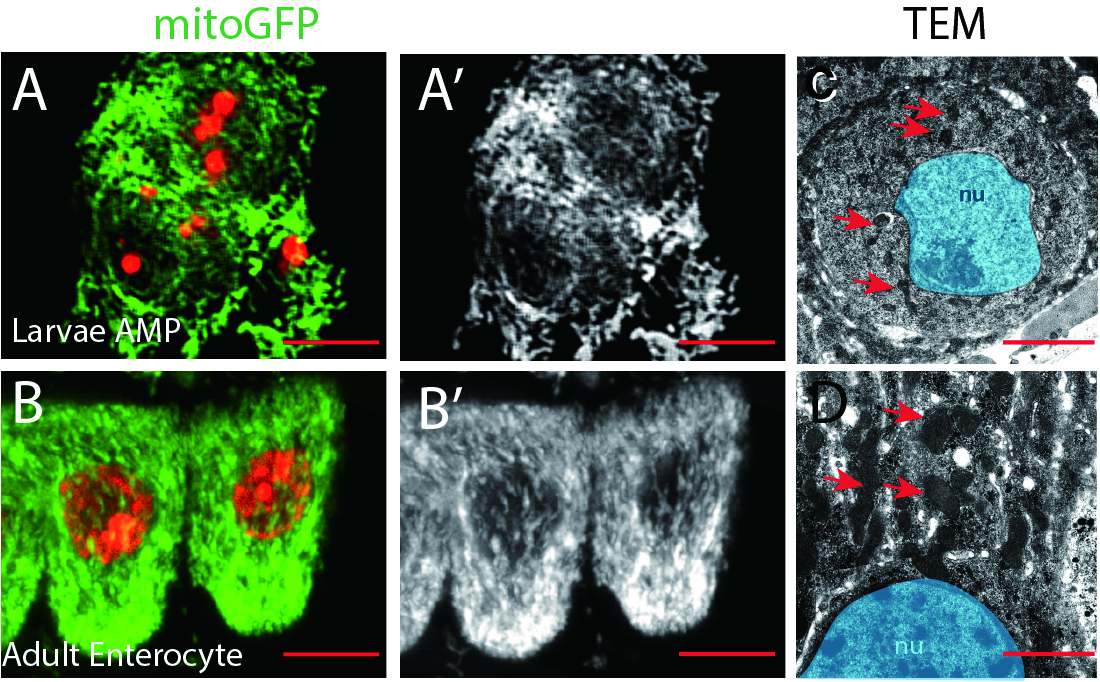


**Figure S3. Proliferation of hindgut progenitors in *byn*-GAL4> *opa1* RNAi flies is largely unaffected**

(A-B) The nuclei of stem cell “niche” was labeled by Wg-LacZ in red and Stat-GFP labeled the progenitor cells in green. The brackets indicated the progenitor cells.

(C-D) In situ hybridization of CG31607, which was transcribed exclusively in progenitor cells as indicated by brackets. Scale bar: A-B, 40µm, C-D,100µm.


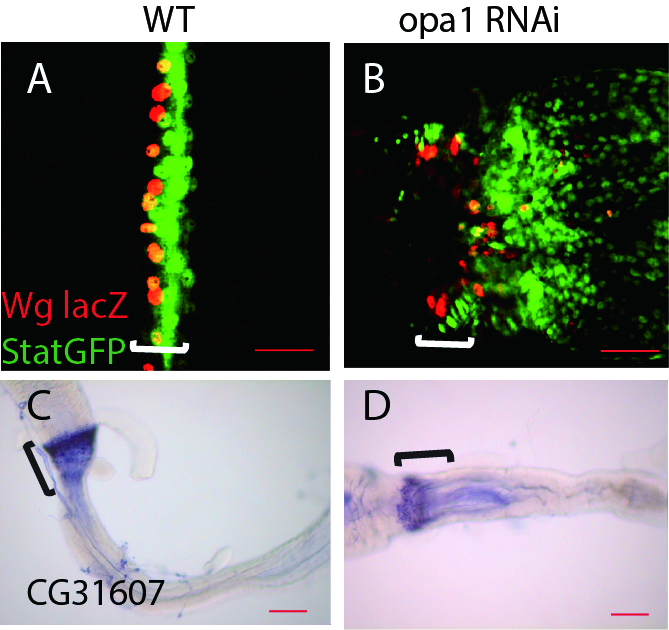


**Figure S4. Hindgut defects in *opa-1*(RNAi) flies are not caused by apoptosis of differentiated enterocytes**

(A-C) Apoptotic nuclei were stained by TUNEL in red. *Stat*-GFP (in green) labeled progenitor cells. Ectopic apoptosis was induced by *UAS* hid, *UAS* rpr in hindgut in C.

(D-E) P35 over-expression (OE) fails to rescue opa-1RNAi hindgut defects including short hindgut and expansion of stat*-*GFP. Stat-GFP (in green) labeled progenitor cells. TOTO-3 labeled nuclei in blue. Scale bar:100µm.


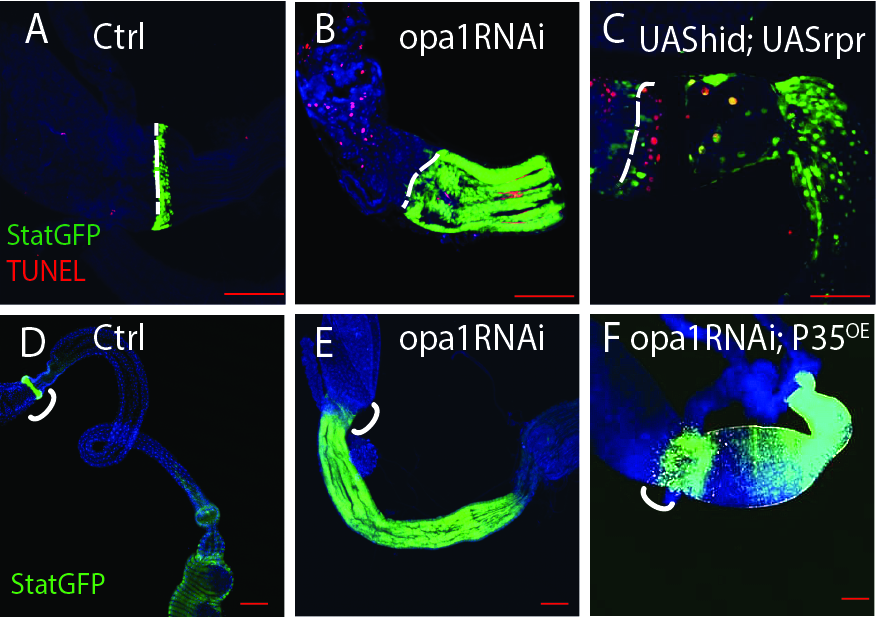


**Figure S5. Mitochondrial membrane potential in hindgut by TMRE staining**

Mitochondria were visualized with mitochondrial targeted mito-GFP driven by BynGAL4.

(A and B) Mitochondrial membrane potential in HPZ (A) and enterocytes (B). B’ and C’ are mito-GFP channels, B’’ and C’’ are red channels with mitochondria stained by TMRE. The dashed lines mark the putative boundary between midgut and hindgut. Note the colocalization of mito-GFP and TMRE, and much stronger TMRE signal in hindgut enterocytes. Scale bar: 20um


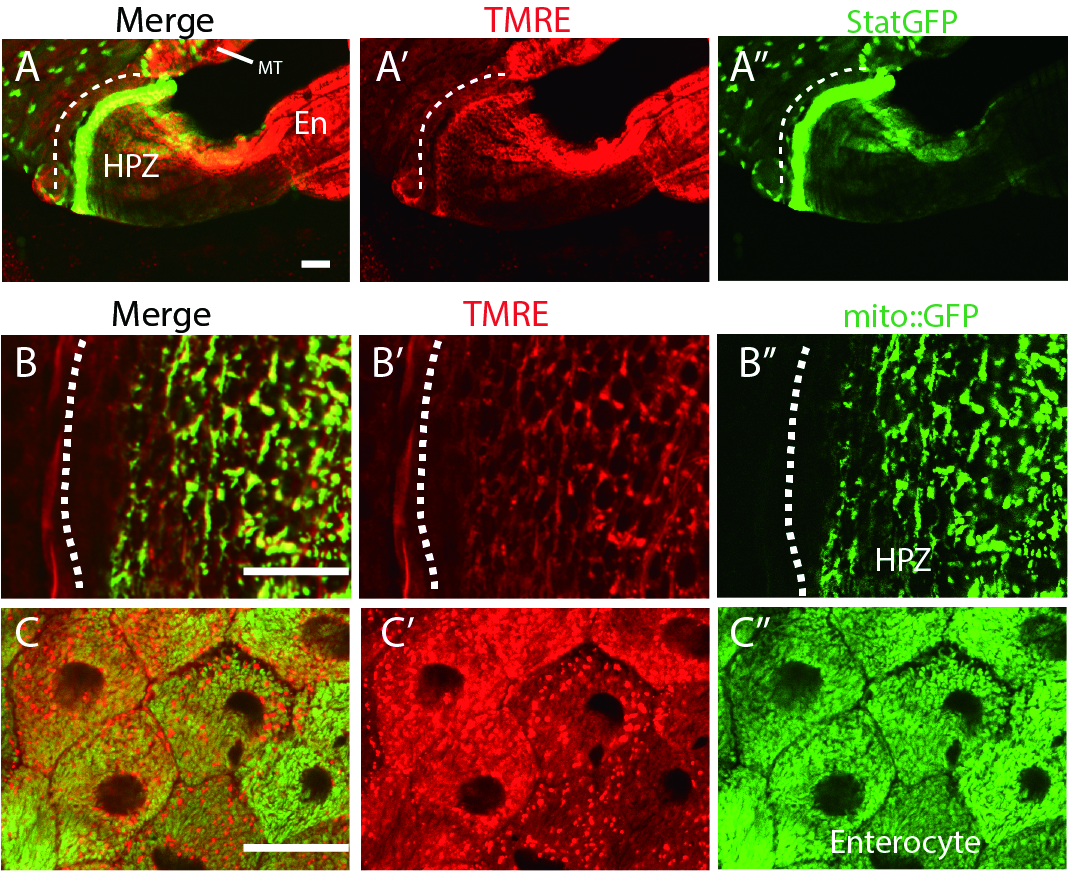


**Figure S6. Differentiation defects of AMP in larvae midgut by *opa1* RNAi**

(A) Schematic view of larvae AMP cluster. Peripheral cells (PC) are crest in shape and labeled in green. Esg positive center cells (CC) are inside the cluster.

(B-E) Differentiation failure of peripheral cells in *opa1*RNAi AMP clusters rescued by *drp1*RNAi. *Esg* positive cells was labeled by *myr-*RFP in cell membrane (in red) and Stat*-*GFP (green) localized outside the islet. In *opa1* RNAi AMP cluster, no typical peripheral cells are found (C). These defects can be rescued by *drp1* RNAi (D). cc is brief for center cells and pc is brief for peripheral cells. Scale bar: 50µm.


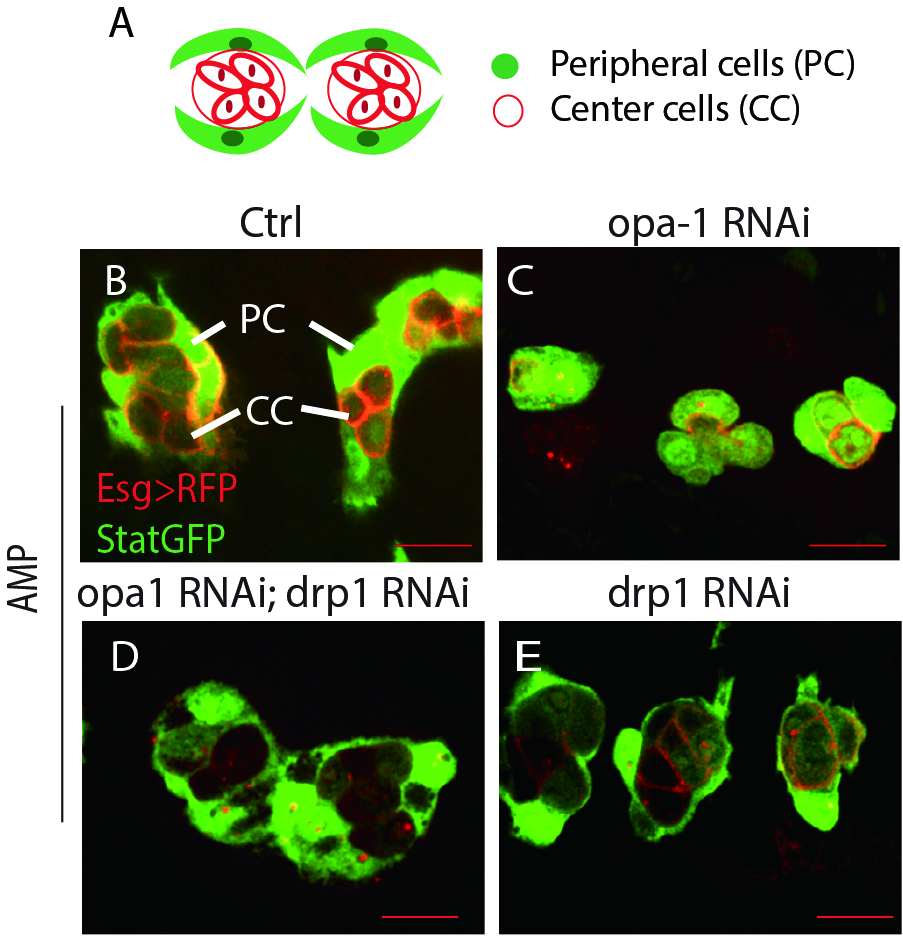


**Figure S7. Ectopic expression of Delta in *opa1* RNAi midgut**

(A) Delta is exclusively expressed in esg positive midgut stem cells in wild type. A’ and A’’ are channels for Delta and Esg respectively.

(B) Massive expansion of Delta and Esg positive cells in *opa1* RNAi midgut. B’ and B’’ are channels for Delta and Esg respectively.

Genotype: *esg-*GAL4, *tub*GAL80^ts^, *UAS*GFP/*opa1* RNAi.

Larvae were shifted from 20°C to 29°C in 2^nd^ instar and the adult midguts were stained with anti-*Delta* antibody. Scale bar: 50µm.

**
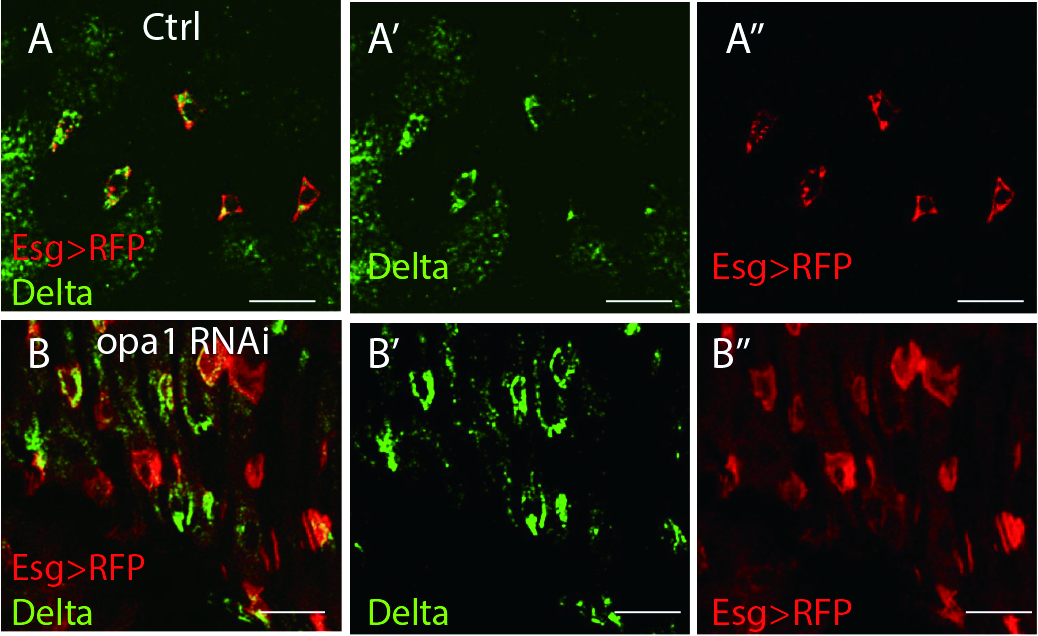
**
